# The impact of CSR on green purchase intention: Empirical evidence from the green building Industries in Taiwan

**DOI:** 10.3389/fpsyg.2022.1055505

**Published:** 2022-11-30

**Authors:** Yi-Tien Tao, Min-Der Lin, Asif Khan

**Affiliations:** ^1^Department of Environmental Engineering, National Chung Hsing University, Taichung, Taiwan; ^2^Department of Marketing and Distribution Management, College of Management, National Kaohsiung University of Science and Technology, Kaohsiung, Taiwan

**Keywords:** corporate social responsibility, green purchase intention, green word of mouth, green attitude, green concern, green trust, sustainable career development

## Abstract

**Introduction:**

Worldwide supplies are being utilized at a disturbing rate and to a significant degree, the building business has been accountable for this ecological deprivation, particularly because of its elevated level of energy expenditure. Hence, this research paper targets the customers of green building industries in Taiwan and developed a framework based on Carroll’s corporate social responsibility (CSR) model, theory of planned behavior (TPB), and cognitive consistency theory (CCT), to measure the impact of CSR on green purchase intention (GPI). Furthermore, it analyzes the impact of CSR on green word of mouth (GWOM), green attitude (GA), green concern (GC), and green trust (GT). Moreover, it explores the roles of GA, GC, GWOM, and GT on GPI. Finally, to study the mediating roles of GC, GA, and GWOM, with the relationship between CSR and GPI.

**Methods:**

The data for this study was collected from 600 customers of green building businesses located in Taiwan with the help of a convenience sampling technique.

**Results:**

As per the results of this research, CSR was discovered to have a positive impact on GPI, GWOM, GA, GC, and GT. Furthermore, GWOM had a significant impact on GPI. Moreover, GC and GT were in a significant relationship with GPI. Consequently, all the relationships were significant except the relationship between GA and GPI. Furthermore, GT, GWOM, and GC fully mediate the relationship between CSR and GPI. However, GA was not found to significantly mediate the relationship between CSR and GPI.

**Discussion:**

The findings of this study provide practical implications regarding the impact of CSR on GPI and the trending issue of sustainable career development.

## Introduction

Global supplies are being utilized at a disturbing rate as a result of Man’s overexploitation (Khan et al., 2021b), and the outcome is not merely rising greenhouse gas discharge, that is altering the worldwide environment for the worse, but similarly declining forestry, failing fisheries, and consuming clean water structures and environmental supplies (Olubunmi et al., 2016). To a considerable degree, the building business has been liable for this environmental deprivation (Dahiru et al., 2012; Dobson et al., 2013; Hussin et al., 2013), particularly because of its elevated level of energy expenditure. Recognizing that the building business will all the time include, to some extent, adverse environmental consequences, green building (GB) has been encouraged and marketed as a leading theory to growth in the construction sector (Olubunmi et al., 2016). It is the GB segment’s reply to endorsing environmental expansion (Dania et al., 2013). GB is the way of designing buildings and employing procedures that are reliable and supply-effective during the project’s life-cycle from location to shape, construction, management, safeguard, and repair (Olubunmi et al., 2016). In the course of strategy and building, green structures employ recycled resources, less water, less energy, and supply effective methods; include a water-sensitive plan and diminish vulnerability to flooding; lessen infecting discharges to air, soil, and water and minimalize light and sound pollution (Li et al., 2014), in this manner reducing harmful effect on the atmosphere (Hussin et al., 2013). Therefore, this ecological-responsive building method has a significant societal and financial impact. Communally, green buildings enhance the livelihood and working atmosphere for individuals. Financially, green constructions put forward cost reductions to holders or occupants (Olubunmi et al., 2016). Also, they are sold or rented at a quicker rate, presenting the prospect of larger earnings (Hwang and Ng, 2013). Hence, it can be indicated that green building is ecologically, financially, and communally beneficial. (Odebiyi Sunday et al., 2010).

Corporations have concentrated on corporate social responsibility (CSR) actions, involving ecological sustainability, individual rights, and public welfare, as a growing amount of clients wish for accountable and sustainable utilization of knowledge (De Grosbois, 2016; Ghaderi et al., 2019; Khan et al., 2021c). Lately, one of the greatest noticeable reproaches on conventional career development models is that these frameworks are inadequate to clarify occupational conduct in the current demanding domain and rapidly-fluctuating work (Asuquo and Inaja, 2013). Capabilities are crucial considerations that offer an exceptional effect on sustainable career development. Nevertheless, the absence of regulations has been challenging to identify and assess these capabilities (Suhairom et al., 2019). Lastly, grounds for instance those of the shareholder method have directed the formation of complete practice-concerned CSR contexts. The foundations describe the important parts of action concerning sustainability and CSR. The elements include market, society, and sustainable career development emerging from the business frameworks, such descriptions are important especially when CSR integrates the collaboration among businesses and the societies in which they work (Nicolopoulou, 2011). Particularly, in the service industry, several corporations have participated in societal accountability programs and employed CSR pursuits as successful promotion instruments (Iyer and Jarvis, 2019). CSR is progressively significant to service corporations as a tactical method since they stimulate clients’ favorite reactions and buying intent by expanding clients’ constructive assessment of a brand (Iyer and Jarvis, 2019; Sung et al., 2020). Investigators have countered CSR programs by trying to determine the effect of CSR on clients’ conduct (Su et al., 2017; Hur et al., 2018). Nevertheless, the conclusions from earlier analyses on CSR are different throughout businesses. For instance, Hur et al. (2018) confirmed that clients’ opinion about CSR has a considerable significant impact on clients’ citizenship conduct. However, Supanti and Butcher (2019) did not discover any substantial impact of CSR on customers’ conduct. The conclusions from earlier findings likewise determined that CSR does not constantly generate encouraging behavior results (Yoon et al., 2006). For instance, employing CSR pursuits could heighten clients’ distrust and pessimistic attitudes (Shim and Yang, 2016). Moreover, Ailawadi et al. (2014) observed the adverse impact of CSR on the share of the wallet. Consequently, extra effort is required to explain the other aspects that might act as mediators among CSR and positive conclusions to boost its advantages (Ahn and Kwon, 2020; Khan et al., 2021a). By implementing CSR methods, a business implies that it pursues earnings and likewise acknowledges worldwide individual beliefs. CSR can expand a business’s image, impact customers’ mindsets in the direction of a company and significantly impact customers’ buzz, buying intents and trustworthiness. Consequently, CSR is tactically critical to a company. The increasing acceptance of CSR methods is further helped by the point that customers are progressively incorporating societal and ecological standards into their expenditure choices. It is a general understanding that CSR impacts customer conduct. Fishbein’s multi-element attitude prototype revealed that customers’ attitudes establish their mindsets that in turn alter their conduct goals (Ajzen and Fishbein, 1975). Consequently, customers’ attitudes regarding CSR decide their feelings about CSR that impacts conduct intents for instance purchase goals. In particular, Mohr et al. (2001) demonstrated that assessments of products, corporations, and buying intents vary on the extent and kind of CSR knowledge supplied. In addition, Gatti et al. (2012) noticed that CSR in combination with apparent quality can offer a competitive benefit by changing buying intents (Lee and Lee, 2015). Hence, this research paper intends to explore the impact of CSR on green purchase intention (GPI).

The study on consumption indicates that this conduct mainly changes due to customers’ demands, attitudes, and priorities and the way they handle these priorities established in their observances. Consequently, in the past thirty years, numerous scientists have presented prototypes for forecasting individual conduct, for instance, the Theory of Planned Behavior (TPB) (Li et al., 2018). Therefore, this investigation intends to discover the influence of CSR on the green attitudes (GA) of clients (Akbari et al., 2019a). Trust is a crucial factor in clients’ judgment-making procedure to make up buying decisions (Jin et al., 2016; Palacios-Florencio et al., 2018). Thus, Palacios-Florencio et al. (2018) suggested that trust is a vital element in creating a lasting association with clients. Hence, this research examines the role of CSR in building the green trust (GT) of customers. (Ahn and Kwon, 2020). Through the increasing agreement in the general public on the degrading atmosphere, which is steadily getting into public sight, for example, supply dearth, and temperature change, the sustainable concern is presently growing to be a popular topic in resolving these ecological and societal-financial difficulties. Therefore, this paper analyzes the impact of CSR on the green concern (GC) (Ye et al., 2020). Furthermore, there is investigational proof concerning the criticalness of the corporation’s CSR endeavors in explaining the word-of-mouth creation procedure. Thus, this paper explores the impact of CSR on green word of mouth (GWOM) (Han et al., 2020a).

This research further intends to measure the relationship between GA (Zhang L. et al., 2018), GT (Ahmad and Zhang, 2020), GC, and GWOM (Zhang L. et al., 2018) on the customers’ GPI. Notwithstanding the significance to examine the part of the interpersonal concepts in the associations between CSR and client’s intent (Inoue et al., 2017), inadequate investigations have analyzed both indirect and direct processes to describe clients’ behavioral intent, particularly in the CSR perspective. Though there are not many theoretical prototypes that employed multiple mediators in the context of CSR, previous CSR studies have mostly employed one mediator for the association between CSR and clients’ conduct (Palacios-Florencio et al., 2018), not examining numerous mediators applying experimental data. To improve the client–corporation association, examining the position of various mediators is important to effectively calculate clients’ behavioral intent (Akrout and Nagy, 2018). Contrasting to earlier research, the recent research explores the mediating roles of GA, GT, GC, and GWOM. Analyzing several interpersonal constructs is better in comparison to analyzing a plain mediation framework as a multiple-mediation framework lets scientists identify every structure concurrently along with the common link among constructs (Hayes and Scharkow, 2013; Ahn and Kwon, 2020).

This research has beneficial theoretical implications for the theory of planned behavior (TPB), cognitive consistency theory, and CSR. The outcomes have significant implications for companies in Taiwan and other developing economies with concerns about green building to enhance the utilization of sustainable services. Taking into consideration customers’ concerns regarding well-being, an applicable sustainable approach is needed to improve that must concentrate on certain customer types, improving customers’ knowledge and understanding of reliable building services. This research covers the following research gaps. First, it measures the impact of CSR on GPI. Second, it analyzes the impact of CSR on GWOM, GA, GC, and GT. Third, it explores the roles of GA, GC, GWOM, and GT on GPI. Finally, it studies the mediating roles of sustainable concern, sustainable attitude, sustainable word of mouth, and the relationship between CSR and sustainable purchase intention. Table 1 signifies the research gaps of the previous studies and indicates the research contribution of this study.

**Table 1:** Previous research contributions.

Research	CSR	PURI	WOM	ATTI	CONC	TRUS
Hajli et al. (2014)			**√**			**√**
Suki (2016)		**√**		**√**		
Zhang L. et al. (2018)		**√**	**√**		**√**	
Zhang B. et al. (2018)		**√**		**√**		
Ahmad and Zhang (2020)		**√**	**√**			**√**
Han et al. (2020b)	**√**	**√**		**√**		
Han et al. (2020a)	**√**	**√**				
Chuah et al. (2020)	**√**				**√**	**√**
Ahn and Kwon (2020)	**√**					**√**
Rausch and Kopplin (2021)		**√**		**√**	**√**	
Chen et al. (2022)		**√**		**√**		
Present Research	**√**	**√**	**√**	**√**	**√**	**√**

CSR, Corporate social responsibility; ATTI, Green attitude; CONC, Green concern; PURI, Green purchase intention; TRUS, Green trust; WORM, Green word of mouth.

## Theoretical background and hypotheses development

### Underpinning the theory

This research study uses Carroll’s CSR model, TPB, and cognitive consistency theory to measure the impact of CSR on GPI. CSR has achieved importance in the industry because CSR can improve companies’ bottom-line productivity and offer a competitive benefit (Abdelhalim and Eldin, 2019). CSR is also an approach that seeks at attaining planned purposes and improving the planet (Khan et al., 2021a). Carroll exhibited CSR in form of a pyramid encompassing four responsibilities comprising economic responsibility, which is associated with the companies profit making regulations; legal responsibility, which is associated with the company’s compliance following the regulations set by the lawmakers; ethical responsibility, which is associated with the companies adherence of fairness and justice; and philanthropic responsibility, which is linked with the companies ability to comply with the activities related to the promotion of social welfare of individuals (Chen et al., 2021). Carroll’s model of CSR is attached to the sustainable development concept, in which societal, financial, and ecological interests are incorporated into corporate strategies (Chuah et al., 2020). Furthermore, the CCT describes the concept that people manage to keep their emotional stability between their insight and assessment toward studies. In other words, people can offset their constructive or pessimistic feelings and develop their behavioral intent consequently. The CCT has been extensively employed to describe persons’ ecological-associated activities for the reason that consumers with encouraging experiences toward ecological-associated methods exhibit constructive GPI (Ahn and Kwon, 2020). Finally, this study also linked TPB with sustainability. Corresponding to the TPB, individuals’ particular actions are defined by the performance of their activities (Ajzen, 2011). TPB has been helpful in forecasting customer intent along with conduct (Shih-Chih and Shing-Han, 2010; Zhang B. et al., 2018; Chen et al., 2022). Hence, this research incorporates TPB in the theoretical model to measure the GPI of customers. Table 2. indicates the operational definitions of the research constructs used in this study.

**Table 2:** Operational definitions of research constructs.

**Constructs**	**Operational definitions**	**References**
**CSR**	CSR is described as a persistent obligation by acting morally in industry and by improving value to financial expansion, living quality, and community. CSR addresses economic, legal, ethical, and philanthropic matters.	Ahn and Kwon (2020)
**PURI**	GPI indicates the probability that customers will purchase a specific product subsequent to their ecological beliefs, and signifies the degree to which customers are willing to acquire products and services from companies with a status for being ecologically responsible.	Newton et al. (2015)
**WOM**	GWOM is the amount to which a customer would advise others regarding constructive ecological communications associated with products and services.	Zhang L. et al. (2018)
**ATTI**	GA about a product is linked to the customers’ inclination and complete assessment of a brand, which personifies their likes and dislikes.	Suki (2016)
**CONC**	GC is a customer’s emotional assessment of ecological concerns and is frequently considered an antecedent to GPI.	Zhang L. et al. (2018)
**TRUS**	GT is the level of customers’ dependence on a particular entity; this trust is based on the faith established from compassion, integrity, and ecological performance.	Ahmad and Zhang (2020)

CSR, Corporate social responsibility; ATTI, Green attitude; CONC, Green concern; PURI, Green purchase intention; TRUS, Green trust; WORM, Green word of mouth.

### Corporate social responsibility and green purchase intention

CSR has been a subject of significance in customer conduct and green industries (Lee et al., 2013; Rashid et al., 2015; Suárez-Cebador et al., 2018). Particularly, CSR has collected substantial consideration from green building specialists and investigators as the ecological decline is growing and developing into an evolving problem (Rashid et al., 2015). Corresponding to Han et al. (2019), CSR signifies a potential influence that a certain corporation does on the ecologically responsible growth of people or society with not much sacrifice concerning its economic operation. This description of CSR is in agreement with Rashid et al.’s (2015) and Han et al.’s (2020a) explanation that a corporation’s supplementary efforts at incorporating the problem of environmental safeguard and interest into its business. A corporation and its pro-ecological environment can stimulate external and internal clients’ constructive mindset for ecologically responsible actions and endorse methods of such actions (Afsar et al., 2020). Once a corporation makes different accountable endeavors for ecological conservation, its workers are motivated to perform environment-friendly behaviors according to the green expectation of the company (Lee et al., 2013). Similarly, once a corporation makes several environmentally friendly CSR efforts, its clients are prone to participate in pro-ecological consumption conduct possessing a significant mindset toward it (Rashid et al., 2015; Han et al., 2019). Unquestionably, a corporation’s CSR actions for the atmosphere significantly indirectly or directly support improving its repute and benefactors’ positive attitude regarding the corporation’s services (Han et al., 2020b). Consequently, the following hypothesis is postulated.

*Hypothesis 1*: There is a significant relationship between CSR and GPI.

### Corporate social responsibility and green word of mouth

A green building industry’s CSR endeavors as recognized by its clients are crucial in describing their intent creation and post-acquisition actions (Tian et al., 2011; Kim and Ham, 2016; Su et al., 2017; Han et al., 2020b). For instance, Su et al. (2017) stated that clients’ opinions of CSR are an immediate predictor of a company’s repute and client fulfillment. Furthermore, Wang and Han (2017) discovered that a corporation’s CSR actions are a crucial element impacting clients’ intellectual assessments of the corporation’s operations and behavioral objectives. Additionally, Tian et al. (2011) acknowledged that clients’ word of mouth and opinions of the CSR of a company help them possess constructive feelings and opinions about the company. In addition, Seo et al. (2015) likewise contended that CSR usually encourages company operations and impacts clients’ practical service value and feelings (Han et al., 2020a) that improve to create a great degree of confidence and loyalty to the corporation and its services (Kim and Ham, 2016). More recently, Han et al. (2020a) discovered that a corporation’s business environmental obligation is a substantial factor in developing client emotional assessment of its services and encouraging constructive conduct for the corporation. Once a particular company is involved in CSR activities related to the environment, customers are expected to evaluate the corporation’s service in a positive direction (Wang and Han, 2017) to have a constructive impact on the company (Tian et al., 2011) and to participate in encouraging actions in the direction of the company (Han et al., 2020b). Consequently, the following hypothesis is postulated.

*Hypothesis 2*: There is a significant relationship between CSR and GWOM.

### Corporate social responsibility and green attitude

CSR is the combination of legal, environmental, economic, and ethical factors in the decision-making procedure associated with the construction of a green service (Gras-Gil et al., 2016; Akbari et al., 2019a, 2019b). Creators have, thus, to support their work with strategies, choices, and activities that are attractive according to the attitudes and principles of the population (Carroll, 2008). Recently, CSR has drawn awareness in the fields of construction and promotion strategies (Öberseder et al., 2014; Alvarado-Herrera et al., 2017; Wei et al., 2018) as it might substantially influence customers’ inclinations and behavioral preferences (Boccia and Sarnacchiaro, 2018). Concerning the worries and doubts concerning green consumption, understanding CSR is especially crucial for the research of customers’ GAs and goals for these services. Nevertheless, there is a shortage of findings on this subject. Pino et al. (2016) utilized Carroll’s (1991) four-dimensional CSR framework to research the impact of CSR on customers’ GAs and intent to utilize green services. Carrolls’ framework comprises four obligations including the economic responsibility, which is related to the delivery of corporations’ services in an environmental-friendly manner, the legal responsibility related to the company’s obligation of following the rules and laws, ethical responsibility related to following the necessary ethical obligations, and philanthropic responsibility related to company’s accountability in terms of benefitting the society and other companies. Though, Pino et al. (2016) explained that only experiences of green businesses manufacturers’ legal and philanthropic responsibilities significantly influence customers’ green for green consumption and their aims to recognize such services correspondingly. Moreover, Martínez and Del Bosque (2013) recognized the significant impact of CSR on customers’ belief in a particular company. Consequently, to further examine the position of CSR in green consumption, in this research, CSR is employed having four dimensions, including legal responsibility, economic responsibility, philanthropic responsibility, and ethical responsibility, in the TPB to examine its impact on customers’ GAs in such services. Consequently, the following hypothesis is postulated.

*Hypothesis 3*: There is a significant relationship between CSR and GA.

### Corporate social responsibility and green concern

CSR is an effective approach that is comprehended generally as a corporation’s responsibility to expand long-term financial, social, and ecological welfare through corporate procedures, strategies, and reserves (Ye et al., 2020). CSR has altered from a philanthropic element to compulsory and required over years (Carroll, 2008) and become a popular business framework that offers a relative gain in several characteristics and encourages company roles to GC (Behringer and Szegedi, 2016; de Sousa Jabbour et al., 2020). CSR has several explanations (Dahlsrud, 2008), however, when it describes GCs, the principle of CSR is established on environmental, social, and economic dimensions (Zhou et al., 2019) that were in accord with the Triple Bottom Line approach. As understood, the philosophy of CSR and GC in the long-term and the equilibrium among the social, environmental, and economic components do coincide. Nevertheless, GC and CSR are unique in their major aims. Behringer and Szegedi (2016) contended that GC aims further on meeting demands; ethical principles; societal, financial, and ecological elements; civil rights; and collaboration, while CSR concentrates on environmental and societal interrelations; shareholder attitude; moral conduct; and volunteering. Correspondingly, Dyllick and Muff (2016) contended that GC concentrates on a macro level, as CSR concentrates solely on industry-level approaches and environmental efficiency. Recently, the collaboration between CSR and GC has increased both practically and theoretically (Behringer and Szegedi, 2016). Hence, there is a growing tendency in the way business has added to GC (Heikkurinen and Bonnedahl, 2013; Behringer and Szegedi, 2016; Engert et al., 2016; Sunder and Prashar, 2020; Van der Waal and Thijssens, 2020). The research areas of sustainability and CSR have been expanding for more than three decades (Fatma and Rahman, 2015), though the addition of both subjects is relatively less and newer. Owing to some components of CSR linked to the sustainable concern of either company, atmosphere, or community, the notion of CSR and its connection to sustainable concern is certainly misinterpreted (Ebner and Baumgartner, 2006). There is yet a few discussions on the association between CSR and GC (Behringer and Szegedi, 2016; Ye et al., 2020). Consequently, the following hypothesis is postulated.

*Hypothesis 4*: There is a significant relationship between CSR and GC.

### Corporate social responsibility and green trust

Corporations have concentrated on CSR endeavors, involving ecological sustainability, social rights, and societal well-being, since a growing amount of clients wish for accountable and sustainable utilization practices (Ghaderi et al., 2019). Particularly, in the service industry, several corporations have participated in societal responsibility programs and employed CSR pursuits as efficient promotion tactics (Ahn and Kwon, 2020). CSR is gradually essential to service businesses as a tactical methodology since they stimulate clients’ favorite reactions and buying intent by improving clients’ constructive assessment of the direction of a brand (Lee and Lee, 2015; Sung et al., 2020). Trust is an essential element in clients’ choice-making procedure to make up buying choices (Choi and La, 2013; Jin et al., 2016). Moreover, trust is a vital component in developing a longstanding bond with clients (Palacios-Florencio et al., 2018; Ahn and Kwon, 2020). GT is a commitment to be contingent on a brand, service, or product, on the faith or belief stemming from its integrity, compassion, and capability regarding its ecological performance (Chen, 2010). Though such beliefs can be formed by secondary cues, for instance, the real utilization practice of the brand represents the most important component of trust. Furthermore, trust lessens the dangers that a trade partner will act speculatively (Chuah et al., 2020), increases value in trades, and improves the likelihood of buying (Chen and Chang, 2012; Kang and Hur, 2012). Augmented by high degrees of certainty in a corporation’s green performance, CSR actions would lead to preferred degrees of brand incorporation into the customer’s feeling of self, and in succession, encourage GT (Chuah et al., 2020). Additionally, trust is the precursor of optimistic conclusions and additionally the development of the connection. Centered on the cognitive consistency theory (Heider, 1946), people make an effort to sustain their emotional stability by balancing their awareness and assessment. For instance, individuals that have a certain opinion about service industries’ CSR actions are further possible to think that a corporation is trustworthy (Jahn and Brühl, 2019). Venger and Pomirleanu (2018) recommended that customers who build trust toward their positive CSR experience certainly assess the corporation (Ahn and Kwon, 2020). Palacios-Florencio et al. (2018) similarly substantiated that CSR insight improves loyalty *via* trust. In addition, Kim et al. (2015) recognized the mediating position of GT in the association between CSR and business repute. Correspondingly, in the service industry, CSR boosts business repute as significant CSR experience improves the degree of GT and revisit intent in the direction of a business (Ahn and Kwon, 2020). Consequently, the following hypothesis is postulated.

*Hypothesis 5*: There is a significant relationship between CSR and GT.

### Green word of mouth and green purchase intention

Word-of-mouth (WOM) implies oral interaction among customers and other individuals or groups, for instance, networks, service creators, professionals, acquaintances, and families (Chaniotakis and Lymperopoulos, 2009; Zhang L. et al., 2018). WOM can express customers’ pleasurable encounters, also known as positive WOM, and additionally bad encounters *via* criticisms and allegations, also known as negative WOM (Anderson, 1998). Expanding WOM to the ecological area, Chen et al. (2014) suggest that GWOM is the amount to which clients advise their acquaintances, families, and associates regarding the encouraging ecological communications and the ecologically friendly attitude of a company. Once a corporation engages in ecological management, clients might believe well in the company and provide encouraging WOM regarding its sustainable activities. However, individuals are frequently eager to reveal and publicize the flaw instead of discovering and announcing the accomplishments. Thus, if a corporation misinforms its customers *via* greenwashing, the wronged customers will give out word of the wrongdoing and inform, or also discourage, others from utilizing the services (Chen et al., 2014). This might indicate a condition where as soon as a customer is informed of greenwashing, several customers will become further disbelieving and might disagree to acquire and discourage others from using the business’s services, specifically in an age of social media in which knowledge spreads extensively and rapidly (Lim et al., 2016). Therefore, it is believed that greenwashing has an adverse influence on GWOM (Chaniotakis and Lymperopoulos, 2009; Chen et al., 2014; Zhang L. et al., 2018). Furthermore, GWOM is expected to urge customers to shift their consumption inclinations and judgments (Zhao and Xie, 2011; Zhang L. et al., 2018). GWOM is crucial for customers’ intentions and several corporations implement it as an efficient promotion tactic (Yang et al., 2012). GWOM has a significant impact on customers’ purchase intentions as individuals have a tendency to make judgments by implying knowledge, which decreases the vagueness of their purchase intentions (Chen et al., 2014). China is experiencing a massive societal revolution, which will certainly impact society’s beliefs, principles, and conduct, and will cause further severe asymmetric data. Keller and Fay (2012) suggest that constructive GWOM can lead to a great degree of integrity so customers are expected to make buying choices whenever they perceive others concerning encouraging knowledge regarding utilization of services. Whenever customers are confused regarding green services or products, possessing better GWOM are further expected to earn the confidence of customers and improve their green purchasing intentions (Chen et al., 2011; Zhang L. et al., 2018). Consequently, the following hypothesis is postulated.

*Hypothesis 6*: There is a significant relationship between GWOM and GPI.

### Green attitude and green purchase intention

Attitude concerning a corporation is related to the customers’ inclination and general assessment of a company that exemplifies their dislikes and likes (Solomon et al., 2014; Suki, 2016). According to a study customers’ GA while acquiring green services was found to be the most important element in forecasting their motive to utilize green services other than the impact of, or references from relatives, networks, and associates (Honkanen and Young, 2015). Similarly, a survey of Greek customers also produced comparable results (Fotopoulos and Krystallis, 2002). Previous researchers observed that buying choices are generally centered on the customers’ GA (Felix and Braunsberger, 2016). Thoughts and constructive repute are the basic consequences that shape client GA and influence their intent to buy sustainable services or products (Thøgersen et al., 2015). Earlier green promotion findings have suggested that customers’ GA concerning environmentally friendly conduct considerably affects their ecological expertise and GPI (Aman Abad et al., 2012; Suki, 2016). Likewise, Yadav and Pathak’s (2016) investigation produced a similar outcome that contended that customers’ GA substantially impacts their sustainable buying intention. Corresponding to these conclusions, another research study stated that GA Indian customers substantially forecast their buying intent of a sustainable service or product (Rana and Paul, 2017). Furthermore, research by Mostafa (2009) demonstrated that customers with constructive GA are further prone to progress a greater tendency to buy green services or products. Finally, a study by Teng (2009) also indicated that customers with a constructive GA for a specific corporation have a tendency to possess a significant GPI. Consequently, the following hypothesis is postulated.

*Hypothesis 7*: There is a significant relationship between GA and GPI.

### Green concern and green purchase intention

GC is a person’s consciousness of ecological difficulties in addition to the individual’s commitment to managing the problem (Akehurst et al., 2012). Many studies consider this ecological concern as an indirect or direct originator of customers’ green buying intents (Chen and Lee, 2015; Newton et al., 2015). In reality, customers with a great level of GC are further expected to have a clear feeling of ecological accountability and follow ecologically friendly actions (Biswas and Roy, 2015), for instance, power-saving, and purchasing ecologically-friendly services and products. Furthermore, they can also identify the company’s greenwashing tactics and be conscious of its adverse effects, therefore decreasing the utilization of such services and products (Newton et al., 2015). Additionally, Kwon et al. (2016) consider GC to have a moderating impact on the relationship between external ecological rankings and corporate sustainability awareness. Moreover, the fundamental concept of the coherence between nature and humans directs individuals to give extra consideration to environmental safeguards. Along with the assistance of this notion, in regard to the serious environmental contamination, customers with elevated GC are expected to enforce their sustainable attitudes onto sustainable utilization practices and decrease their inattentive buying goals (Johnstone and Tan, 2015; Zhang L. et al., 2018). Consequently, the following hypothesis is postulated.

*Hypothesis 8*: There is a significant relationship between GC and GPI.

### Green trust and green purchase intention

**Figure 1:**
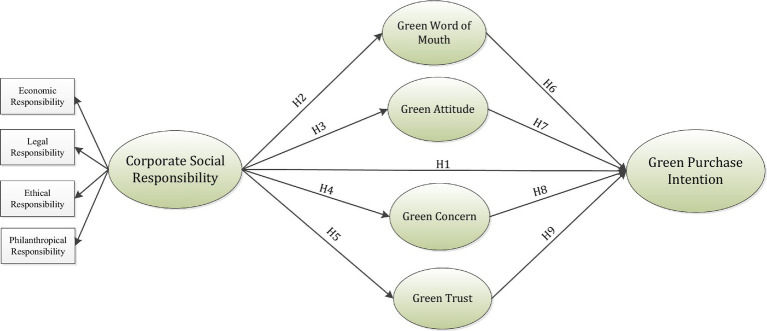
Theoretical framework.

Trust denotes the commitment to acknowledge a threat by subjecting oneself to a certain peripheral component or someone (Ahmad and Zhang, 2020). GT signifies the tendency of customers to be convinced of the services and products that they trust owing to their recognized environmental efficiency (Wang et al., 2018). Furthermore, GT likewise represents the individual expectation, opinions, or beliefs of all behaviors to be ecological and it should similarly associate with individual and ecological concerns not including any damaging effects. Trust in a current structure is dependent on regulations and laws but it is similarly affected by the competency of the service representatives responsible to connect with the clients (Grönroos, 2001). Customers expect that industries will likewise behave in a reliable, communally appropriate, and ethical manner (Hosmer, 1995). Therefore, a greater level of GT between consumers suggests a better possibility of purchasing and utilizing sustainable services and products. GT is deemed a motivator, which directs clients’ green buying in expectation of high-quality services and longstanding relations. In addition, GT is considered an essential construct owing to its possible impact on the apparent customer risk rooted in customer conduct, which allows the customers to assess the effect of specific choices and the comparative benefits earned (Ahmad and Zhang, 2020). Consequently, GT can influence customers’ green buying intention (Chen, 2010). Consequently, the following hypothesis is postulated Figure 1.

*Hypothesis 9*: There is a significant relationship between GT and GPI.

## Methodology

### Operationalization of variable

CSR was measured by the items from Chen et al.’s (2021) study while the items to assess GWOM and GT were taken from Ahmad and Zhang (2020) study. Moreover, GPI was measured by the items recommended by Zhang B. et al. (2018) study. Lastly, items from Rausch and Kopplin’s (2021) study were utilized to measure GA and GC. The Likert scale was used to record the respondents’ approval with respect to a given measurement item. In this scale the level of approval is usually on a 7-point, where 1 (= strongly disagree), 7 (= strongly agree), and 4 relate to a neutral group. A pretest was performed *via* a sample of 80 customers and a response rate of 94.7% was accomplished.

### Data collection and sample

The data for this study was collected from the customers of GB businesses located in Taiwan. This study targets the customers of GB businesses in Taiwan. A research company in Taiwan was employed to gather the data for this research. A convenience sampling technique was used to select 600 customers. A questionnaire was distributed to the customers of GB businesses.

## Results and analysis

### Data analysis

Two methods for computing and estimating partial least squares (PLS) were executed. In the first stage, reliability estimation was performed. The second stage is related to the descriptive analysis and evaluation of the study model. The purpose of the two previously mentioned phases is to verify the reliability of the components, including the confirmation of the links between the variables (Hulland, 1999). The PLS has been adopted and is considered the most excellent means of describing the basic collaboration between variables, thus allowing the management of both framework variables and measurement items (Petter et al., 2007). Furthermore, since PLS has simpler restrictions on changing normality and ambiguity; it is ideal for investigating connections if the variables are irregularly dispersed. Thus, it possesses the benefits of a computational dynamic research framework (Chin et al., 2003; Xu et al., 2021; Zhao and Khan, 2021). Therefore, PLS is more suitable for this analysis than earlier SEM evaluation methods to calculate the relationships between the variables, reduce calculation errors, and avoid collinearity.

### Convergent and discriminant validity

PLS-SEM incorporates both the outer and internal frameworks. Convergent and discriminant reliability was employed to measure the outer framework. According to Table 1, the factor loading values ranged between 0.743 and 0.947. These values were found to be higher than the 0.50 factor loading threshold value (Hair et al., 2014). Hence, the study proved to be having significant individual item reliability. The composite reliability (CR) was used to measure the internal reliability. The CR values were found to be higher than the 0.60 threshold value (Hair et al., 2014). Hence, the internal reliability of the research constructs was achieved (Bagozzi et al., 1991). Furthermore, the AVEs values ranged between 0.686 and 0.810. Consequently, the Average variance extracted (AVE) values of this study were found higher than the threshold value of 0.50 (2014).

The degree of discrimination among analyzing variables and various construct measures is characterized by discriminatory validity. Table 2 implies a decent discriminant validity for every construct, by indicating that the factor loading value of every item is the highest in the latent structure as compared to other structures (Hair et al., 2016).

Tenenhaus et al. (2005) equation was employed to calculate the goodness of fit (GOF) for this study. The quality of the research framework is analyzed as follows:


$$GOF=\sqrt{\bar{AVE}}\;x\;\sqrt{\bar{R^{2}}}=\sqrt{0.542\;x\;0.717}=0.623$$

Subsequent to the aforementioned result, the GOF is 0.623 and attains the 0.376 cut-off requirements for a substantial impact size (Wetzels et al., 2009).

### Empirical results

Smart PLS was used to analyze the internal path coefficients of this study. The inner model was analyzed and according to researchers, the value of p should be lower than the threshold value of 0.05. On the other hand, the t-value should be higher than the threshold value of 1.96.

**Figure 2:**
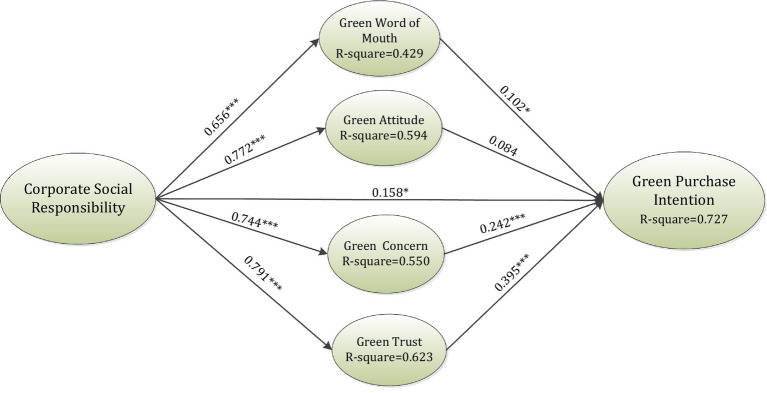
Results of the inner model. ***value of *p* < 0.001, **value of *p* < 0.01, *value of *p* < 0.05.

As per the results of this research, as shown in Table 3 and Figure 2, CSR was discovered to have a positive impact on GPI (*β* = 0.158, *t*-value = 2.112), GWOM (*β* = 0.656, *t*-value = 18.989), GA (*β* = 0.772, *t*-value = 29.952), green concentration (*β* = 0.744, *t*-value = 27.658), and GT (*β* = 0.791, *t*-value = 30.342). Furthermore, GWOM had a significant impact on GPI (*β* = 0.102, *t*-value = 2.224). Moreover, GC (*β* = 0.242, *t*-value = 5.025) and GT (*β* = 0.395, *t*-value = 6.829) were in a significant relationship with GPI. Consequently, all the relationships were significant except the relationship between GA and GPI (*β* = 0.084, *t*-value = 1.482).

**Table 3:** Construct validity and reliability.

**Constructs**	**Indicators**	**Factor loadings**	**Composite reliability**	**Average variance extracted (AVE)**
**Economic responsibility**	ECON1 ECON2 ECON3 ECON4	0.872 0.887 0.756 0.765	0.893	0.677
**Legal responsibility**	LEG1 LEG2 LEG3	0.876 0.924 0.901	0.928	0.810
**Ethical responsibility**	ETHI1 ETHI2 ETHI3	0.867 0.869 0.772	0.875	0.701
**Philanthropic responsibility**	PHIL1 PHIL2 PHIL3	0.853 0.888 0.893	0.910	0.771
**Green attitude**	ATTI1 ATTI2 ATTI3	0.947 0.930 0.931	0.955	0.876
**Green concern**	CONC1 CONC2 CONC3 CONC4	0.938 0.924 0.921 0.933	0.962	0.863
**Green purchase intention**	PURI1 PURI2 PURI3 PURI4 PURI5 PURI6	0.743 0.879 0.823 0.887 0.861 0.765	0.929	0.686
**Green trust**	TRUS1 TRUS2 TRUS3 TRUS4	0.888 0.880 0.859 0.845	0.924	0.754
**GWOM**	WORM1 WORM2 WORM3	0.865 0.868 0.798	0.881	0.713

CSR, Corporate social responsibility; ATTI, Green attitude; CONC, Green concern; PURI, Green purchase intention; TRUS, Green trust; WORM, Green word of mouth.

This study utilized Kofod-Petersen and Cassens's (2005) activity theory to measure the mediation impacts as specified in Table 4. Furthermore, the outcomes of the mediation were also produced by Smart PLS. Corresponding to the outcomes indicated in Table 4, GT (*β* = 0.312, *t*-value = 6.704), GWOM (*β* = 0.066, *t*-value = 2.247), and GC (*β* = 0.181, *t*-value = 4.791) fully mediates the relationship between CSR and GPI. However, GA (*β* = 0.065, *t*-value = 1.474) was not found to significantly mediate the relationship between CSR and GPI.

**Table 4:** Cross loadings.

	**ATTI**	**CONC**	**ECON**	**LEG**	**ETHI**	**PHIL**	**PURI**	**TRUS**	**WORM**
**ATTI1**	0.947	0.598	0.689	0.625	0.537	0.645	0.622	0.629	0.470
**ATTI2**	0.930	0.611	0.669	0.653	0.503	0.637	0.624	0.640	0.452
**ATTI3**	0.931	0.573	0.680	0.619	0.550	0.624	0.645	0.612	0.484
**CONC1**	0.580	0.938	0.618	0.592	0.513	0.658	0.684	0.640	0.437
**CONC2**	0.568	0.924	0.603	0.607	0.510	0.624	0.642	0.638	0.397
**CONC3**	0.617	0.921	0.621	0.557	0.528	0.647	0.699	0.676	0.410
**CONC4**	0.593	0.933	0.660	0.575	0.532	0.648	0.702	0.663	0.434
**ECON1**	0.583	0.530	0.872	0.615	0.603	0.580	0.551	0.561	0.452
**ECON2**	0.597	0.598	0.887	0.691	0.620	0.633	0.566	0.623	0.493
**ECON3**	0.541	0.524	0.756	0.392	0.443	0.563	0.562	0.494	0.435
**ECON4**	0.678	0.567	0.765	0.498	0.468	0.584	0.606	0.645	0.383
**LEG1**	0.732	0.556	0.652	0.876	0.544	0.580	0.528	0.597	0.436
**LEG2**	0.599	0.594	0.613	0.924	0.652	0.562	0.566	0.569	0.436
**LEG3**	0.492	0.542	0.565	0.901	0.667	0.516	0.482	0.539	0.431
**ETHI1**	0.384	0.423	0.480	0.430	0.867	0.490	0.494	0.529	0.661
**ETH2**	0.390	0.404	0.503	0.435	0.869	0.492	0.506	0.547	0.687
**ETHI3**	0.605	0.551	0.630	0.800	0.772	0.609	0.570	0.580	0.497
**PHIL1**	0.595	0.551	0.595	0.502	0.535	0.853	0.617	0.605	0.432
**PHIL2**	0.597	0.648	0.643	0.507	0.522	0.888	0.713	0.634	0.446
**PHIL3**	0.598	0.626	0.647	0.604	0.637	0.893	0.724	0.664	0.572
**PURI1**	0.496	0.619	0.593	0.568	0.599	0.670	0.743	0.721	0.434
**PURI2**	0.606	0.582	0.589	0.454	0.509	0.663	0.879	0.694	0.489
**PURI3**	0.472	0.562	0.556	0.488	0.497	0.597	0.823	0.581	0.511
**PURI4**	0.580	0.586	0.584	0.460	0.533	0.667	0.887	0.668	0.518
**PURI5**	0.590	0.562	0.555	0.392	0.475	0.638	0.861	0.639	0.497
**PURI6**	0.587	0.719	0.541	0.528	0.521	0.634	0.765	0.661	0.430
**TRUS1**	0.648	0.633	0.639	0.549	0.601	0.653	0.694	0.888	0.511
**TRUS2**	0.560	0.638	0.572	0.476	0.540	0.613	0.723	0.880	0.495
**TRUS3**	0.616	0.593	0.655	0.575	0.556	0.647	0.662	0.859	0.454
**TRUS4**	0.502	0.584	0.584	0.592	0.616	0.600	0.703	0.845	0.495
**WORM1**	0.410	0.348	0.426	0.427	0.519	0.459	0.443	0.426	0.865
**WORM2**	0.463	0.374	0.425	0.383	0.567	0.455	0.509	0.460	0.868
**WORM3**	0.394	0.415	0.499	0.411	0.740	0.482	0.509	0.530	0.798

CSR, Corporate social responsibility; ATTI, Green attitude; CONC, Green concern; PURI, Green purchase intention; TRUS, Green trust; WORM, Green word of mouth.

## Discussions

This research paper developed a framework based on Carroll’s CSR model, theory of planned behavior (TPB), and cognitive consistency theory, to measure the impact of CSR on sustainable purchase intention. Furthermore, it analyzes the impact of CSR on GWOM, GA, GC, and GT. Moreover, it explores the roles of GA, GC, GWOM, and GT on sustainable purchase intention. Finally, to study the mediating roles of GC, GA, and GWOM, with the relationship between CSR and sustainable purchase intention (see Tables 5, 6).

**Table 5:** Hypothesis results.

**Hypotheses**	**Path coefficient (*β*)**	***T*-values**	***p*-values**	**Results**
**H1: CSR → PURI**	0.158	2.112	0.035	Supported
**H2: CSR → WORM**	0.656	18.989	0.000	Supported
**H3: CSR → ATTI**	0.772	29.952	0.000	Supported
**H4: CSR → CONC**	0.744	27.658	0.000	Supported
**H5: CSR → TRUS**	0.791	30.342	0.000	Supported
**H6: WORM → PURI**	0.102	2.224	0.026	Supported
**H7: ATTI → PURI**	0.084	1.482	0.139	Not Supported
**H8: CONC → PURI**	0.242	5.025	0.000	Supported
**H9: TRUS → PURI**	0.395	6.829	0.000	Supported

CSR, Corporate social responsibility; ATTI, Green attitude; CONC, Green concern; PURI, Green purchase intention; TRUS, Green trust; WORM, Green word of mouth.

**Table 6:** Indirect effects result.

**Indirect effects**	**Path coefficient (*β*)**	***T*-values**	***p*-values**	**Results**
**CSR → TTI → PURI**	0.065	1.474	0.141	Not Supported
**CSR → TRUS → PURI**	0.312	6.704	0.000	Supported
**CSR → WORM → PURI**	0.066	2.247	0.025	Supported
**CSR → CONC → PURI**	0.181	4.791	0.000	Supported

CSR, Corporate social responsibility; ATTI, Green attitude; CONC, Green concern; PURI, Green purchase intention; TRUS, Green trust; WORM, Green word of mouth.

According to the results of this study, CSR was discovered to have a positive impact on GPI. The results were somewhat similar to research conducted by Lee and Lee (2015). Their research intended to recognize the impact of Chinese customers’ attitudes regarding aspects of CSR on self-congruence and their buying intents in the fashion business. Furthermore, according to the results of this study CSR was in a significant relationship with GWOM, and GA. These findings were somewhat similar to previous studies (Akbari et al., 2019a; Han et al., 2020a). Additionally, CSR was found to be in a significant relationship with GCs. This finding was somewhat similar to a previous study (Ye et al., 2020). Also, CSR and sustainable trust were discovered to be in a significant relationship. These findings were in accordance with a study conducted by Ahn and Kwon (2020). Their study intended to assess the way hotels can efficiently affect clients’ investment activities by leveraging general clients’ CSR insight, trust, and loyalty (Ahn and Kwon, 2020).

Furthermore, GWOM had a significant impact on GPI. The finding was somewhat similar to a previous study by Zhang L. et al. (2018). Their study studied the customers’ greenwashing experiences impact their sustainable buying intents by incorporating the mediating position of GWOM and the moderating position of GC. Likewise, GC and GT were in a significant relationship with GPI. The results were in accordance with previous studies (Zhang L. et al., 2018; Ahmad and Zhang, 2020). Consequently, all the relationships were significant except the relationship between GA and GPI. This result was not found to be in accordance with previous studies (Suki, 2016).

## Theoretical implications

This research has beneficial theoretical implications for the theory of planned behavior (TPB), cognitive consistency theory, and CSR. Furthermore, as a result of the increasing competition between service industries, there is a requirement to examine the way corporations can improve the efficacy of advertising pursuits, which are crucial for constant development (Ahn and Kwon, 2020). Earlier findings also indicated a significant impact of CSR on clients’ behavioral intent in other service industries, for instance, banking industries (Hur et al., 2018). Furthermore, the outcomes of this study imply that the cognitive consistency theory can assist scientists to comprehend the method of faith, feelings, and conduct in the CSR narrative. The findings of this research additionally help the earlier study (Supanti and Butcher, 2019) that did not discover the significant impact of CSR on behavior consequences. By employing the cognitive consistency theory, the suggested framework backed the important theory of fundamental CSR conduct that customers are encouraged to manage their beliefs, emotions, and behaviors in a significant manner. The outcomes described GT as an essential factor in the decision procedure to sustain a person’s constructive psychological equilibrium. Hence, CSR can generate a significant connection between clients and corporations by developing their trust. Here, the existing research increases the theoretical understanding regarding cognitive consistency theory by explaining that relational constructs are vital to harmonize clients’ awareness, emotions, and conduct. In the CSR framework, corporations should produce trust by creating trustworthy relations with clients. Since there are inadequate analyses that investigated several mediators of the association between CSR purchase intention in green building service industries’ CSR perspective, this research improves the knowledge of the antecedents regarding GPIs (Ahn and Kwon, 2020).

## Practical implications

The outcomes have significant implications for companies with concerns about green building to enhance the utilization of sustainable services. Taking into consideration customers’ concerns regarding well-being, an applicable sustainable approach is needed to improve that must concentrate on certain customer types, improving customers’ knowledge and understanding of reliable building services. Green building is a credible service, customers might not understand whether manufactured utilizing reliable or dangerous techniques without them being informed beforehand. Consequently, customers’ awareness and understanding regarding green buildings have a substantial part in making buying associated choices. Moreover, the research can be beneficial for the green building industries to recognize their target customers by displaying the impact of considerations on green building utilization purchases. Distinct and precise marketing tactics must be applied for various customers to enhance the buying intention and commitment to give a premium price for green building services. Moreover, this research offers recommendations and ideas for regulators that sustainable construction brands should be accredited for the market. Adequate evidence concerning green building services must be supplied to customers across various mass media. Additionally, to provide green building services to customers, green construction tools and techniques must be implemented (Zhang B. et al., 2018). Moreover, clients are not expected to produce constructive behavior reactions even if they possess constructive CSR awareness. Thus, green building marketing professionals should constantly examine if their CSR strategies efficiently convey their impacts to society in general and green building customers. For instance, green building industries might be able to follow clients’ responses regarding CSR activities by organizing an online analysis or advocating hashtag experiences *via* social media. Urging clients to contribute their ideas regarding CSR projects might be the greatest example of knowing their perceptions about CSR initiatives (Hur et al., 2018). Also, green building industries must pay consideration to providing CSR communications to clients as an effective way of enhancing constructive clients’ attitudes for instance commitment and trust in the corporation. Clients should be notified that the corporation’s CSR actions are authentic and genuine (Park et al., 2014) with the intention that CSR pursuits can improve clients’ degree of trust. Furthermore, in terms of the rules and regulations related to the human resource management (HRM) perspective, and the circumstances in which they are beneficial in enabling the procedures of sustainable career development and CSR. Likewise, it essentially combines the conceptualization of job characteristics as a crucial component that impacts the routine of information transmission by internationally successful experts (Nicolopoulou, 2011).

## Limitations and future research

Even though this research has added valuable practical understandings to the green building-oriented CSR narrative, some limitations should be recognized. First, this study was performed with the help of a survey method. To recognize the causal association between CSR and psychological status, potential investigators can perform investigational analyses by controlling the kind of CSR pursuits and data. Second, this research only centered on CSR activities in green building industries in a developing economy. The green building industry is further concentrated on ecological characteristics of CSR than other characteristics, for instance, societal aspects in developed economies. Hence, future researchers can modify and employ the research framework in developed economies to get novel results. Lastly, potential scientists must contemplate other mediators when they employ the cognitive consistency theory in the association between CSR insights and GPIs, to acquire more valuable insights regarding the green building industries in the CSR context.

## Data availability statement

The raw data supporting the conclusions of this article will be made available by the authors, without undue reservation.

## Author contributions

Y-TT and M-DL: conceptualization. AK: formal analysis. Y-TT: investigation. Y-TT, M-DL, and AK: writing—original draft and writing—review and editing. All authors contributed to the article and approved the submitted version.

## Conflict of interest

The authors declare that the research was conducted in the absence of any commercial or financial relationships that could be construed as a potential conflict of interest.

## Publisher’s note

All claims expressed in this article are solely those of the authors and do not necessarily represent those of their affiliated organizations, or those of the publisher, the editors and the reviewers. Any product that may be evaluated in this article, or claim that may be made by its manufacturer, is not guaranteed or endorsed by the publisher.
